# Medical Bibliography. A. & B.

**Published:** 1834-04-01

**Authors:** 


*Medical Bibliography. A & B.* By James Atkinson, Surgeon to H.R.H. the late Duke of York., Senior Surgeon to the York County Hospital, and the York Dispensary, &c.—London, 1834. 8vo. pp. 380 and viii.

This is the commencement of a list of medical writers and their works, with remarks and critiques. Some of these are sensible enough, while others are so strangely facetious, that they remind us rather of a village wag, who would rub a sleeper's face with burnt cork, or pull away the chair from an expectant sitter, than of a senior surgeon to a county hospital. Take a couple of examples.

"Alcmaeon, Crotoninensis. If this author was the disciple of Pythagoras, in the 35th age of the world, or about 497 years before Christ, as is reported, it well becomes me to beg pardon of his manes, for not having already introduced him.

"He is mentioned as being the first veterinary surgeon who wrote on the anatomy of animals, and was possibly a relation of the famous Milo, of Croton, who could bear a bull (I don't say bull and bear, 'ne quid nimis!') upon his shoulders. Milo might have been the apprentice of Alcmaeon; and now and then, pro re nata, in the way of his profession, have had occasion to carry a sick bull into the surgery to his master. Let us here observe, in a parenthesis, how surprisingly one trifling incident of history may clear up another, 'and the sons of Alcmaeon shall never repine.'" (P. 39.)

"Questions simple as these always console me for my deficiency or direct stupidity in regard to mathematics, alias Mathew's mad tricks; and (faute de l'emportement) such they do appear to me. I dare not even hint that the mathematician and the conjuror are synonymous, lest possibly, some one of the mathematical galaxy might affix one branch of his compass into my sinciput, (not sense-pot,) and by concentric circles, or elliptic crossings with the other, scoop out the poor pittance of brain, which stingy nature obviously, has given to me. If such however were done, oh, what a figure I should cut; for I still venture to reckon on my having so many brains as to induce me to consider a man as nothing without brains. And I will further presume on the fact, (miserable dictû !) calling Oxford and Cambridge to witness, that he who had been the first in order of mathematics has sometimes dwindled to be the last in the scale of nature. Quo te dementia!" (P. 161.)

The following is much better, though certainly out of place:

"There is not perhaps any science wherein so many monstrous good jokes and superlative romances are exhibited as in the science of medicine. For instance: it is a monstrous good joke in any practitioner to suppose that his patients will always abide by him; and yet it is a common error. It is a monstrous good joke, to be observed, that men, of the first science and longest practice, cannot frequently gain the confidence of their patients; when a Merry Andrew doctor and his mountebank can take a town by storm in a moment; nay, can persuade the mob, mobility, and nobility, (as well as the gens de lettres,) that they have actually cured them of diseases which they never had. It is a monstrous good joke, apparently to affect, that arsenic shall poison one man, who was well; and as quickly cure another, who was ill. It is a monstrous good joke, to perceive, in a town, that a man who came to that town without any character, (because he had not any to bring,) shall, to a moral certainty, supplant, in a very short time, by hook or by crook, every other man there, who had one. But this monstrous good joke is practised, and with success, every day. It is a monstrous good joke, that a medical man, who is confessedly without brains, shall contrive completely to suck the brains of another, who has not brains to perceive it. It is a monstrous good joke, to observe, sometimes, that the best informed practitioner in a town shall seldom get a fee; whilst the greatest medical fool in the town shall seldom miss one." (P. 325.)

The editions of Aretæus are far more numerous than they are often supposed to be: we can afford room only for a part of the enumeration.

"EDITIONS IN 1700.

"*Pataviæ*, 8*vo*. 1700, Eloy; Haller deems it imperfect.
4*to*. 1719, with Boerhaave's Greek text, Crassus's Latin version, and by J. Groenvelch.

"Clarendon. Typ. *Oxon. fol. formâ majori*, 1723, } Gr. et Lat. Ætiologica, Semeiotica, et Therapeutica, sive, &c. Wigan's edition.

"This most perfect and beautiful edition is in chartis maximis, and is produced from a survey of the most elaborate and impartial texts, codices, and translations. It has the advantage of Michael Mattaire's Tract de Aretæi dialecto, or the Ionic dialect used by him, with the Lexicon; it is the first specimen of any Greek medical author from the Clarendon press, (only 300 copies,) and is rated by authors 'splendidissima, accuratissima, plenissima,' or, as we Roman catholics superlatively express, 'a plenary indulgence.'

"Kestner, however, observes of this edition, and it is worth notice, 'optandum tamen foret, versionem hanc non rejectam esse sub calcem græci textûs, sed aut ad latus ejus positam, aut eidem subjectam saltem,' as being very useful to fellows, I don't imply of the universities, nor you, nor me, 'quique sine cortice natare norunt,' who can swim without bladders? Kestner delays giving an opinion of the comparative merits of the Oxford edition, because he had never obtained a sight of it; and, similarly, I was a long while before I could get a sight of his book. This Oxford edition was collated with the Harleian, and Vatican proofs, codices, or editions.

"For the editions, &c. of Aretæus, see Wigan, and pay particular attention to his preface.

"*London*, 4*to*. 1726, three first books with Petit's Comm. by Mattaire.

Pet. Vander Aa, *Lugd.Bat. fol. major*, 1731, } De Causis et signis Acut. et Diut. Morb. Lib. iv. Gr. et Lat. notis Var. —as Boerhaave, Scaliger, Triller, Pet. Petit, Wigan, Mattaire; cum Junii Pauli Crassi vers. Latin. è recognitione G. M. T., subjectá.

"Dr. Clarke observes that this edition is not so elegant, but more useful than the Oxford edition. The extremes of beauty and of use are seldom combined. It contains the whole of Petit's Comm. It is an excellent edition.

"Ianson Vander Aa, *Lugd. fol.* 1735, } by Boerhaave, from Goupylius's Greek edition of 1554, and the same as 1731.

"This is the most copious and complete Greek edition. But, as Boerhaave does not enumerate this edition of his own, it is merely a new titled edition, by rogues of booksellers. Rogues of booksellers—impossible!

"See British Journal of 1751, for observations on some singular diseases of Aretæus and Cœlius Aurelianus." (P. 18.)

Our author informs us that he does not know German, which he calls "most odontolgoid and difficult," and he consequently seldom mentions German books in his list; when he does, their titles are sadly mangled: thus, one of Blumenbach's works is entitled Geschiete der Knocken, instead of Geschichte der Knochen; and, instead of Ueber den Bildungstrieb, we have Uber den bil dungstrict. (P. 324.) At p. 161 we have Avenbrugger's treatise on Percussion thus mentioned: "Vienna, 8vo. 1781, De percussione Thoracis —Inuentum novum ex." Without pretending to guess at the meaning of this, we will just observe, that the first edition of Avenbrugger's treatise is dated 1761, and that its title is "Leopoldi Auenbrugger, Medicinæ Doctoris, in Cesareo Regio Nosocomeo nationum Hispanico medici ordinarii. Inventum novum ex percussione thoracis humani ut signo abstrusos interni pectoris morbos detegendi. Vindobonæ. MDCCLXI."

In reviewing such a literary oddity as the volume before us, we may stand excused if we are infected by its example, and mention the dedication last: it is a very choice bit. "To all idle medical students in Great Britain, sit sacrum." We have been obliged (hard fate!) to content ourselves with the *word* sacrum: not so our author; his dedication is engraved, and he has the thing itself, so that it constitutes, probably for the first time, an exquisite rebus.

